# Commentary: Gender differences in job satisfaction and work-life balance among Chinese physicians in tertiary public hospitals

**DOI:** 10.3389/fpubh.2025.1545743

**Published:** 2025-03-27

**Authors:** B. Sreya, Meghna Rajan, T. Balamurugan, B. Abirami

**Affiliations:** ^1^School of Entrepreneurship & Management, Joy University, Tirunelveli, Tamil Nadu, India; ^2^Department of Commerce, PSG College of Arts and Science, Coimbatore, Tamil Nadu, India

**Keywords:** job satisfaction, work-life balance, gender difference, hospitals, China

## 1 Introduction

In comprehensive research, Liu et al. (1) examine the complex interplay of gender, job satisfaction, and work-life balance (WLB) among Chinese physicians in tertiary public hospitals. With a substantial and representative sample size, this study challenges prevailing perceptions of gender inequalities within the healthcare sector and highlights culturally sensitive workplace dynamics. This commentary aims to expand on the findings and discuss implications for broader healthcare policy and gender equity initiatives framed within existing literature.

### 1.1 Research context and significance

The research addresses an under-researched area by focusing on gender disparities in two critical aspects of professional life: satisfaction and balance between work and life. The study stands out due to its extensive participant base (*N* = 22,128) and the diverse range of hospitals involved, providing a panoramic view of the issue in the Chinese context. Prior studies on job satisfaction and WLB have often revealed gender-based differences, particularly within healthcare settings (2, 3). The prevalence of dual-income households among Chinese medical professionals may play a role in reducing women's domestic burdens, potentially weakening traditional gender roles in healthcare settings. Future studies should investigate whether this factor contributes to the observed gender similarities in job satisfaction and WLB.

### 1.2 Methodological strengths and limitations

The study's methodological rigor is commendable. Validated tools, such as the Minnesota Satisfaction Questionnaire (MSQ), ensure that results are reliable and replicable. The decision to incorporate a simplified yet effective measure of WLB is particularly noteworthy, aligning with established psychometric approaches in similar studies (4). However, some limitations are present, including the cross-sectional design, which does not establish causation, and reliance on self-reported measures that could introduce bias (5). Additionally, the study may have potential survivorship bias due to its focus on tertiary public hospitals. Future research should examine whether rural and primary-care settings exhibit different gender disparities in job satisfaction and WLB.

## 2 Findings and implications

Contrary to widespread assumptions about gender disparities, Liu et al. (1) report that male and female physicians in China have similar levels of job satisfaction and WLB. This finding contrasts with previous studies that identified significant gender-based differences, particularly in cultures with traditional gender roles (6). One possible interpretation is that workplace policies and evolving social attitudes may be contributing to a more balanced professional experience across genders. However, this does not necessarily indicate that gender disparities have been eliminated. Factors such as institutional policies, workload distribution, and external societal expectations may still play a role in shaping job satisfaction and WLB.

Moreover, the study's focus on healthcare settings raises questions about how these findings may translate to other sectors. Workplace policies and socioeconomic factors in healthcare may create unique conditions that influence gender equality differently compared to high-stress industries such as technology and finance. Future studies should explore whether similar gender dynamics exist in these sectors and whether insights from healthcare can inform broader gender equity strategies (7).

## 3 Recommendations for future research

While the study offers robust data, it also suggests opportunities for more in-depth research. In review, conducting longitudinal studies could be beneficial in tracking changes over time to identify causal relationships and observe trends in work-life balance and job satisfaction. Additionally, employing qualitative research methods, such as in-depth interviews, could provide richer insights into the personal and professional challenges faced by healthcare professionals across various demographics (8). Expanding research beyond tertiary hospitals could further illuminate how these dynamics manifest in diverse healthcare settings, ranging from rural clinics to urban specialized centers. Furthermore, cross-cultural studies that examine similar metrics in different nations might reveal potential global patterns or cultural specificities, as highlighted by Mueller et al. (9).

## 4 Discussion

These findings contribute to the broader discourse on workplace gender equity. While no significant differences in job satisfaction and WLB were found between male and female physicians, this does not mean gender disparities have been fully addressed. Instead, these patterns may reflect the impact of evolving workplace policies and socio-economic factors in China (10). Further research is needed to examine variations across medical specialties and healthcare settings, as policy changes, cultural shifts, and economic factors likely shape these outcomes (11). Comparative studies across professions and regions could provide deeper insights into gender dynamics in healthcare.

## 5 Conclusion

The study offers significant insights into the relationship between gender, job satisfaction, and work-life balance, demonstrating progress toward greater equality in healthcare settings in China. The findings emphasize the importance of addressing modifiable factors, such as working hours and income, rather than solely depending on gender-specific strategies. As gender dynamics continue to change, ongoing research and policy updates are essential to promote fairness and improve job satisfaction in the workplace.
